# Prognostic value of VISTA in solid tumours: a systematic review and meta-analysis

**DOI:** 10.1038/s41598-020-59608-w

**Published:** 2020-02-14

**Authors:** Xin-Lin He, Ying Zhou, Huan-Zi Lu, Qun-Xing Li, Zhi Wang

**Affiliations:** Guanghua School of Stomatology, Guangdong Provincial Key Laboratory of Stomatology, Stomatological Hospital, Sun Yat-Sen University, Guangzhou, 510055 People’s Republic of China

**Keywords:** Cancer, Immunology

## Abstract

In the last few years, V-domain Ig-containing suppressor of T cell activation(VISTA) has been reported as a prognostic biomarker in articles including various solid tumours. However, their conclusions have been controversial. For this reason, we performed this meta-analysis to further verify the prognostic value of VISTA in solid tumours. All relevant literature was identified from PubMed, Embase, the Cochrane Library and Web of Science. Ten studies, including 2, 440 patients, were eligible for the analysis. The pooled results showed that high expression of VISTA was associated with favourable overall survival (OS) than that seen with low expression of VISTA (7 studies, hazard ratio (HR) = 0.75, 95% confidence interval (CI): 0.66–0.86, *P* < 0.001). In addition, high expression of VISTA significantly correlated with high numbers of CD8 (+) tumour infiltrating lymphocytes (TILs) (3 studies, risk ratio (RR) = 1.80, 95% CI: 1.41–2.31, *P* < 0.001). In conclusion, these results indicate that VISTA is a potential prognostic biomarker in solid tumours.

## Introduction

V-domain Ig-containing suppressor of T cell activation (VISTA), also known as PD-1H, is a member of the immunoglobulin (Ig) superfamily, whose extracellular domain bears homology to the B7 family ligand programmed death ligand 1 (PD-L1). In both mice^1^ and humans^2^, VISTA is predominantly expressed on granulocytic, myeloid cells and T cells. Similar to some members of the B7-CD28 family^3^, T cells both express and respond to VISTA^4^. *In vitro* proliferation assays, VISTA suppressed the proliferation of both CD4 (+) and CD8 (+) T cells. In mouse tumour models of methylcholanthrene 105-induced fibrosarcoma, Wang^1^ found that overexpression of VISTA on tumour cells accelerated tumour growth. More recently, Le Mercier^5^ reported that a VISTA monoclonal antibody synergized with a tumour vaccine to impair the growth of established B16-BL6 tumours. These studies suggest that VISTA is a novel negative checkpoint regulator.

Recently, a number of studies have reported the expression of VISTA in the tumour microenvironment of human solid tumours, and these studies found that VISTA was a potential prognostic biomarker associated with patients’ survival. However, their conclusions were controversial. Villarroel^6^ found that high levels of VISTA correlated with better overall survival (OS) in non-small-cell lung cancer (NSCLC) than low levels of VISTA. Loeser’s study^7^ showed the same trend in esophageal adenocarcinoma (EAC). In contrast, Kuklinski’s study^8^ showed that high expression of VISTA was associated with poor disease-specific survival (DSS). Therefore, further research is needed to better illustrate the prognostic value of VISTA.

In this study, a systematic review of the eligible literature on this topic was performed by us using the PubMed, Embase, the Cochrane Library and Web of Science databases. Then, a meta-analysis of the survival rates (including OS, DSS and tumour-specific survival (TSS)) of patients expressing different levels of VISTA was conducted. Our results indicated that high expression of VISTA, compared with low expression of VISTA, correlated with better OS and high numbers of CD8 (+) tumour infiltrating lymphocytes (TILs).

## Results

### Characteristics of the included studies

**Figure 1:**
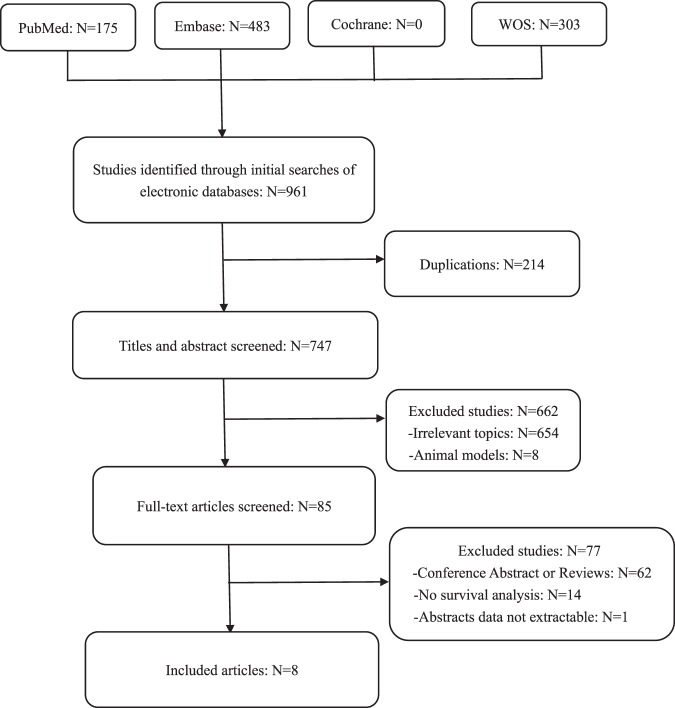
Flow diagram of studies identifed, included, and excluded.

As presented in Fig. 1, a total of 8 articles^6–13^, including 10 studies and 2,440 patients, met the inclusion criteria for the meta-analysis. The main characteristics of the included studies are shown in Table 1. The publish year of all included articles was between 2017 and 2019. The Newcastle–Ottawa Scale^14^ (NOS; Supplementary Table 1) was used to assess the included studies. Approximately one-third of the studies were conducted in Asia (n = 3). The remaining studies were conducted in North America (n = 4) and Europe (n = 3). Cancer types included hepatocellular carcinoma (HCC), oral squamous cell carcinoma(OSCC), NSCLC, ovarian cancer (OC), primary cutaneous melanoma(PCM), gastric cancer(GC), EAC and malignant pleural mesothelioma(MPM). The sample size of the included studies ranged from 65 to 464. The expression level of VISTA in included studies is presented in Supplementary Table 2. Among the 10 studies, 7 studies explored the prognostic value of VISTA in terms of patients’ OS, 2 studies explored the TSS, and 1 study explored the DSS. As far as we understand, the data regarding TSS and DSS can be combined into one group.

**Table 1:** Characteristics of the included studies.

Author and year	Country	Ethnicity	Cancer type	Sample size	Gender M/F	Cut-off value	Detection method	TNM stage	Outcome	HR estimation	Study design	NOS score
Zhang 2018^12^	China	Asian	HCC	183	157/26	≥5%	IHC	I-IV	OS	reported	P	7
Wu 2017^11^	China	Asian	OSCC	165	NA	>median	IHC	I-IV	OS	estimated	P	6
Villarroel 2018^6^	USA	Caucasian	NSCLC Cohort 1	324	287/37	>median	IF	I-IV	OS	estimated	P	7
Villarroel 2018^6^	USA	Caucasian	NSCLC Cohort 2	292	139/153	>median	IF	I-IV	OS	estimated	P	7
Liao 2018^10^	China	Asian	OC	65	NA	Score ≥ 5	IHC	I-IV	TSS	reported	P	7
Kuklinski 2018^8^	USA	Caucasian	PCM	85	49/36	>0	IHC	I-IV	DSS	reported	P	7
Boger 2017^9^	Germany	Caucasian	GC	464	289/175	>34IC/mm^2^	IHC	I-IV	TSS	estimated	P	6
Loeser 2019^7^	Germany	Caucasian	EAC Cohort 1	158	115/43	>4%	IHC	I-IV	OS	estimated	P	7
Loeser 2019^7^ Muller 2019^13^	Germany USA	Caucasian Caucasian	EAC Cohort 2 MPM	393 311	353/40 NA	>4% >40%	IHC IHC	I-IV I-IV	OS OS	estimated reported	P P	7 6

NA: not available, HCC: hepatocellular carcinoma, OSCC: oral squamous cell carcinoma, NSCLC: non-small cell lung cancer, OC: ovarian cancer, PCM: primary cutaneous melanoma, GC: gastric cancer, EAC: esophageal adenocarcinoma, MPM: malignant pleural mesothelioma, OS: overall survival, DSS: disease-specific survival, TSS: tumor-specific survival, IHC: immunohistochemistry, IF: immunofluorescence, P: prospective, NOS: Newcastle–Ottawa Quality Assessment Scale, HR: hazard ratio.

### Methodological quality of the included studies

The quality of the included studies was generally high. All of the studies had an independent assessment of outcome. The majority of the included studies had long enough follow-up durations (5 years), and most of them provided an adequate follow-up. However, none of these eligible studies had representativeness of the exposed cohort because the study objects were patients selected from the hospital. In the majority of the studies, methods for handling intention-to-treat analysis and missing data were not described adequately.

### High expression of VISTA in solid tumours is associated with favourable OS

**Figure 2:**
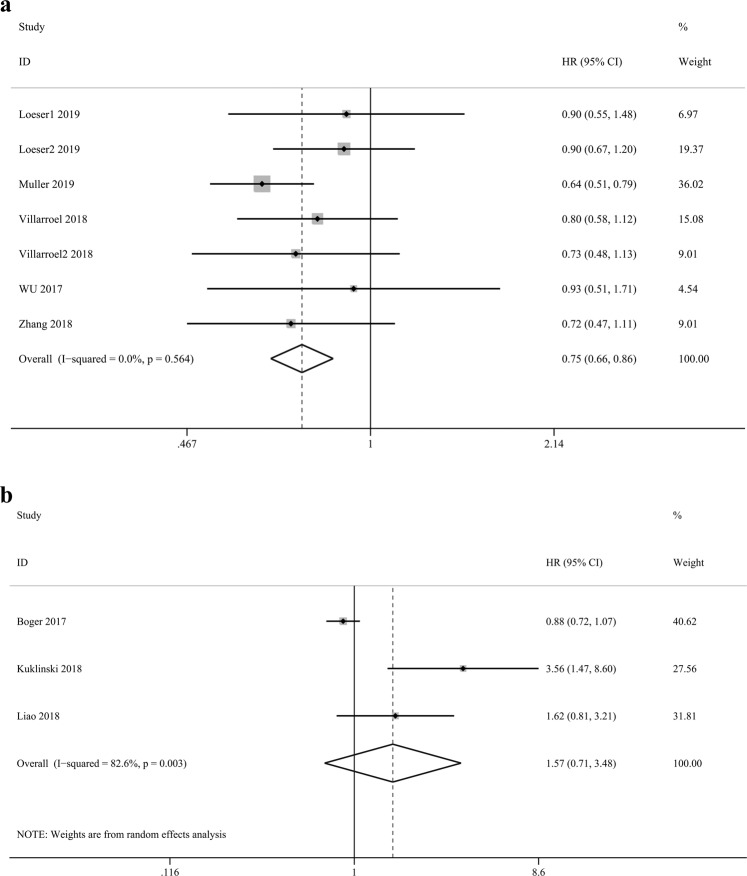
(**a**) High expression of VISTA in solid tumours is associated with favourable OS. (**b**) Forest plot of the association between VISTA and patients’ DSS(or TSS).

Seven studies^6,7,11–13^ reported the relationship between the expression of VISTA and patients’ OS. We applied a fixed effect model to calculate the pooled hazard ratio (HR) and 95% confidence interval (CI) because there was no obvious heterogeneity (*P* = 0.564, I^2^ = 0.0%). The results showed that high expression of VISTA was associated with better OS than low expression of VISTA. (HR = 0.75, 95% CI: 0.66–0.86, *P* < 0.001, Fig. 2a).

### The prognostic value of VISTA in patients’ DSS(or TSS)

Three studies^8–10^ reported the relationship between the expression of VISTA and patients’ DSS (or TSS). A random effect model was applied to calculate the pooled HR and 95% CI due to obvious heterogeneity (*P* = 0.003, I^2^ = 82.6%). However, no obvious trend in DSS (or TSS) was found according to the expression of VISTA (HR = 1.57, 95% CI: 0.71–3.48, *P* = 0.268, Fig. 2b).

### High expression of VISTA correlates with high numbers of CD8 (+) TILs

**Figure 3:**
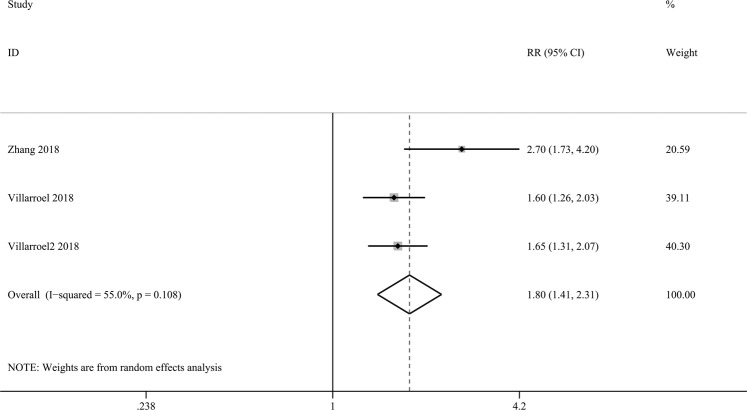
High expression of VISTA correlates with high numbers of CD8 (+) TILs.

To further analyse the possible factors that may affect the prognostic value of VISTA in solid tumours, we researched the correlation between the expression of VISTA and patients’ clinicopathological characteristics. Interestingly, we found that high expression of VISTA was significantly associated with high numbers of CD8 (+) TILs (risk ratio (RR) = 1.80, 95% CI: 1.41–2.31, *P* < 0.001, Fig. 3). As shown in Table 2, no obvious trend in TNM stage or gender was found according to the expression of VISTA.

**Table 2:** Correlation of the expression of VISTA and clinical features.

Variables	Studies	RR	95% CI	Model	Heterogeneity I^2^ (%)	*P* Value
Gender	6^6,7,11,12^	1.11	0.98–1.26	Fixed	0.0	0.10
TNM stage	7^6,7,9,11,12^	0.95	0.78–1.14	Random	56.0	0.57
CD8 + TILs	3^6,12^	1.80	1.41–2.31	Random	55.0	<0.001

RR: risk ratio CI: confidence interval TILs:tumor infiltrating lymphocytes.

### Sensitivity analysis and publication bias

**Figure 4:**
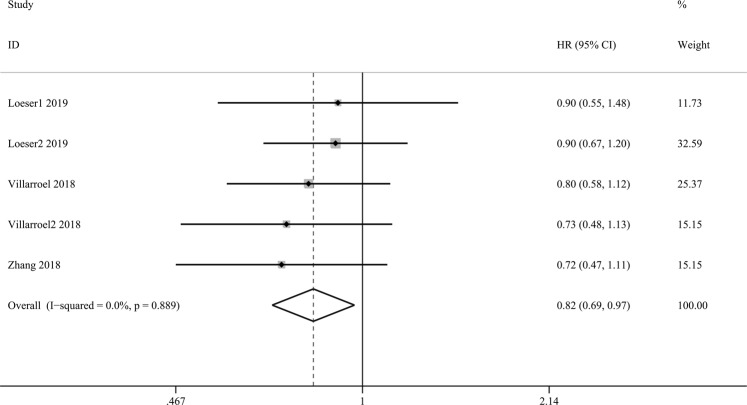
Overall forest plots of sensitivity analysis for the association between VISTA and OS.

Studies with high NOS scores (≥7) were included in the sensitivity analysis of the association between the expression of VISTA and patients’ OS. As shown in Fig. 4, there was no change in the trend regarding HR and 95% CI. High expression of VISTA correlated with a better OS than low expression of VISTA in the high NOS scores studies. According to the guideline, publication bias should not be assessed when the included studies are less than 10, so we did not evaluate the publication bias.

## Discussion

As far as we know, this is the first study to systematically evaluate the prognostic value of VISTA in human solid tumours. Our results revealed that high expression of VISTA was associated with better OS. To further analyse the possible factors that may affect the prognostic value of VISTA in solid tumours, we investigated the correlation between the expression of VISTA and patients’ clinicopathological characteristics. The results indicated that high expression of VISTA significantly correlated with high numbers of CD8 ( + ) TILs.

In the last few years, a number of molecules, such as PD-L1, programmed death 1(PD-1), lymphocyte activation gene 3(LAG3) and T cell immunoglobulin- and mucin-domain-containing molecule-3(TIM3) have been considered as negative immune checkpoints. In 2014, Lines^2^ first defined VISTA as a new negative immune checkpoint. *In vitro* proliferation assays, VISTA suppressed the proliferation of both CD4^+^ and CD8^+^ T cells. In mouse models, VISTA blockade decelerated tumour growth, and overexpression of VISTA accelerated tumour growth. As described above, it seemed that VISTA was associated with poor prognosis. However, Villarroel^6^ found that high levels of VISTA correlated with better OS in NSCLC. Coincidentally, Loeser’s study^6^ showed the same trend in esophageal adenocarcinoma. Moreover, we found the same trends not only in the studies of VISTA, but also in some studies of other negative immune molecules, such as PD-1 and PD-L1^15–20^. Badoua showed that PD-1^+^ TILs were associated with favourable OS in human papillomavirus(+) head and neck cancer. Similarly, Zhu’s study demonstrated that high expression of PD-L1 on tumour cells correlated with better OS than that seen in nasopharyngeal carcinoma patients with low expression of PD-L1. The reasons that may account for this discrepancy include biological differences between mice and humans, different types of tumours and the subjective evaluation of immunohistochemistry (IHC) or immunofluorescence (IF) scores.

Recently, an increasing number of researchers^21–29^ have paid great attention to the CD8 (+) TILs in the tumour microenvironment due to their anti-tumour effect. Gabrielson’s research^21^ illustrated that high numbers of CD8 (+) TILs significantly correlated with a low rate of recurrence and prolonged relapse-free survival in patients with HCC. Goode^22^ showed that in high-grade serous ovarian cancer, high numbers of CD8 (+) TILs were significantly associated with improved OS. Ye^28^ found that high numbers of CD8 (+) TILs predicted favourable prognosis in lung adenocarcinoma patients. These studies may indicate that CD8 (+) TILs are a prognostic biomarker that correlates with a favourable prognosis in solid tumours. In our study, we found that high expression of VISTA was remarkably associated with high numbers of CD8 (+) TILs. This result may partly account for the conclusion that high expression of VISTA correlates with favourable OS in solid tumours.

In conclusion, VISTA may act as a positive prognostic biomarker in solid tumours. Nevertheless, due to the current rarity of studies of the association between the expression of VISTA and patients’ prognosis in solid tumours, we cannot assess publication bias. Therefore, more studies are required for further analysis.

## Methods

### Literature search strategy

We searched all relevant literature in Embase, PubMed, the Cochrane Library and Web of Science on September 28^th^, 2019, without language restriction. These databases were thoroughly searched by the following strategy: ((((((((((VISTA[Title/Abstract] OR VSIR[Title/Abstract]) OR B7H5[Title/Abstract]) OR GI24[Title/Abstract]) OR B7-H5[Title/Abstract]) OR Dies1[Title/Abstract]) OR PD-1H[Title/Abstract]) OR SISP1[Title/Abstract]) OR PP2135[Title/Abstract]) OR C10orf54[Title/Abstract])OR DD1alpha[Title/Abstract]) AND (((cancer[Title/Abstract] OR tumor[Title/Abstract]) OR tumour[Title/Abstract]) OR carcinoma[Title/Abstract]). Two investigators (X.L.H. and Y.Z) independently checked all eligible articles. They reached an agreement via mutual discussion to solve any discrepancies.

### Inclusion criteria

Studies eligible for inclusion met the following rules:(1) the studies detected the expression of VISTA in human solid tumor by IHC or IF; (2) the association of the expression of VISTA and OS/DSS/TSS in solid tumours was reported; (3) HR and 95% CI were reported or could be extracted in the articles or supplementary materials; (4) articles were published as research papers.

### Exclusion criteria

The exclusion criteria included the following: (1) the articles were conference abstracts or reviews; (2) full text of the article was not available; (3) the studies used animal models.

### Data extraction and quality assessment

Information was independently extracted by two researchers (H.Z.L. and Q.X.L.) from the included studies. The collected information included the name of first author, publication year, study country, cancer type, sample size, gender, detection method of the expression of VISTA, cut-off value, TNM stage, and outcome including OS and DSS(or TSS). Two investigators (H.Z.L. and Q.X.L.) independently estimated the HRs and 95% CIs of survival data via Cox univariate analysis. When the articles only provided Kaplan-Meier curves, Engauge Digitizer 4.1 and spreadsheets provided by Tierney^30^ and Parmar^31^ were used to estimate the HRs and 95% CIs. When there were discrepancies, another investigator (X.L.H.) participated in the process. Two investigators(H.Z.L. and Q.X.L.) evaluated the included studies’ quality independently by the NOS(scores: 0–9), and we defined studies that had high NOS scores (≥7) as high-quality. The investigators reached an agreement by mutual discussing when there were inconsistent results.

### Statistical analysis

The association between the expression of VISTA and patients’ prognosis was evaluated by meta-analysis by collecting data from all included studies. We calculated outcome endpoints including OS, DSS and TSS via pooled HRs and 95% CIs. HRs > 1 indicated a poor prognosis. The correlation between the expression of VISTA and the clinicopathological characteristics was evaluated by pooled RRs and 95% CIs. Cochrane’s Q statistic and the I^2^ statistic were used to assess the heterogeneity among the included studies. A random effects model was used to calculate pooled HRs and 95% CIs when there was substantial heterogeneity (Q test: *P* < 0.1 or an I^2^ > 50%). If not, a fixed effects model was used. Studies of high quality(NOS scores ≥ 7) were selected for sensitivity analysis. RevMan 5.3 and Stata 12.0 statistical software (Stata Corporation, College Station, TX, USA) were used to perform all statistical analyses. A difference was considered significant with a two-tailed p < 0.05.

## Supplementary information

Supplementary Information.
