# Supplementary material for: Prognostic value of VISTA in solid tumours: a systematic review and meta-analysis
Source: Sci Rep. 2020 Feb 14;10:2662. doi: 10.1038/s41598-020-59608-w (PMC7021832; doi:10.1038/s41598-020-59608-w)
Supplement: Supplementary file 1 — Supplementary Information. [file 41598_2020_59608_MOESM1_ESM.pdf]

Prognostic value of VISTA in solid tumours: A Systematic Review and Meta-analysis

Xin-Lin He[1,*],Ying Zhou[1,*], Huan-Zi Lu[1], Qun-Xing Li[1],Zhi Wang[1]

[1]Guanghua School of Stomatology, Guangdong Provincial Key Laboratory of Stomatology, Stomatological Hospital, Sun Yat-Sen University, Guangzhou, 510055, PRC.

[*]These authors contributed equally.

Correspondence to:Zhi Wang(wangzh75@mail.sysu.edu.cn)

Supplementary Table 1. Risk of bias in prospective studies based on the modified Newcastle-Ottawa Scale.

Study	***SELECTION*** Representativeness of the Exposed Cohort	Selection of the Non-Exposed Cohort	Ascertainment of Exposure	Demonstration That Outcome of Interest Was Not Present at Start of Study	***COMPARABILITY*** Comparability of Cohorts on the Basis of the Design or Analysis	Assessment of Outcome	***OUTCOME*** Long Enough Follow-Up for Outcome to occur	Adequacy of Follow-Up of Cohorts	Total
Zhang 2018	0	1	1	1	1	1	1	1	7
Wu 2017	0	1	1	1	1	1	0	1	6
Villarroel 2018	0	1	1	1	1	1	1	1	7
Villarroel 2018	0	1	1	1	1	1	1	1	7
Liao 2018	0	1	1	1	1	1	1	1	7
Kuklinski 2018	0	1	1	1	1	1	1	1	7
Boger 2017	0	1	1	1	1	1	1	0	6
Loeser 2019	0	1	1	1	1	1	1	1	7
Loeser 2019	0	1	1	1	1	1	1	1	7
Muller 2019	0	1	1	1	1	0	1	1	6

Supplementary Table 2. Expression level of VISTA in included studies

Study	VISTA high	VISTA low
Zhang 2018	Staining score of ≥5% IC or TC	Staining score of <5% IC or TC
Wu 2017	≥median score	<median score
Villarroel 2018 Cohort1	≥median score	<median score
Villarroel 2018 Cohort2	≥median score	<median score
Liao 2018	IS≥5	IS < 5
Kuklinski 2018	Staining(+) in TIICs	No staining in TIICs
Boger 2017	>34ICs/mm2	≤34ICs/mm2
Loeser 2019 Cohort1	>4% staining of lymphocytes	1-4% staining of lymphocytes
Loeser 2019 Cohort2	>4% staining of lymphocytes	1-4% staining of lymphocytes
Muller 2019	>40% staining of TCs	≤40% staining of TCs

IC:immune cell,TC:tumour cell, IS: immunoreactivity score, TIIC:tumour-infiltrating inflammatory cell
